# Monthly Record of Deaths in the City of Buffalo

**Published:** 1856-07

**Authors:** C. L. Dayton

**Affiliations:** Health Physician


ART. XI.—*Report of Mortality in Buffalo for the month of May*, 1856. By C. L. DAYTON, M. D., Health Physician.

| DISEASES. | No. | Males. | Females. |
|---|---|---|---|
| Accidens, | 3 | 3 | |
| " by taking poison, | 1 | 1 | |
| " Asphyxia Immersorum, | 1 | 1 | |
| Apoplexia, | 2 | 1 | 1 |
| Asphyxia, | 1 | 1 | |
| Abscess Spinalis, | 1 | 1 | |
| Cholera Infantum, | 2 | 1 | 1 |
| Cynanche Trachealis, | 1 | | 1 |
| Diarrhœa, | 1 | | 1 |
| Dentitio, | 3 | 2 | 1 |
| Delirium Tremens, | 2 | 1 | 1 |
| Debilitas, | 2 | 1 | 1 |
| Eclampsia, | 10 | 4 | 6 |
| Enteritis, | 4 | 2 | |
| " Typhoides, | 1 | 1 | |
| Erysipelas Phlegmanodes, | 1 | 1 | |
| Febris Typhoid, | 1 | 1 | |
| " Congestiva, | 1 | | 1 |
| " Typhus, | 1 | 1 | |
| " Scarlatina, | 1 | | 1 |
| Fungus Hæmatodes Ulerina, | 1 | | 1 |
| Fracture Cranii, | 2 | 2 | |
| Hydrocephalus, | 5 | 3 | 2 |
| Hæmoptisis, | 1 | 1 | |
| Hepatitis, | 1 | 1 | |
| Hydrops, | 3 | | 3 |
| Hyperæmia Cerebralis, | 1 | 1 | |
| Leucorrhœa, | 1 | | 1 |
| Morbus Cordis, | 2 | 1 | 1 |
| " Encephalosis, | 1 | 1 | |
| Meningitis, | 1 | 1 | |
| Peritonitis, | 1 | 1 | |
| " Puerperarum, | 1 | | 1 |
| Pneumonitis, | 6 | 4 | 1 |
| Paralysis, | 1 | 1 | |
| Phlebitis Uterina, | 1 | | 1 |
| Pertussis, | 2 | 1 | 1 |
| Rheumatic Pericarditis, | 1 | 1 | |
| Scrophula, | 1 | 1 | |
| Senectus, | 4 | 2 | 2 |
| Still-born, | 7 | 3 | 2 |
| Syphilis, | 1 | | 1 |
| Suicide, by hanging, | 1 | 1 | |
| Tuberculosis Pulmonalis, | 17 | 9 | 8 |
| Variola, | 1 | | 1 |
| Ignotus, | 19 | 11 | 8 |
| Total, | 123 | | |

### REGISTER OF MORTALITY—Continued.

### SEXES.

| | |
|---|---|
| Males, | 72 |
| Females, | 48 |
| Sex not given, | 3 |
| Total, | 123 |

### AGES.

| | | | |
|---|---|---|---|
| Still-born, | 7 | Between 10 years and 20 years, | 2 |
| 1 day, | 3 | " 20 " " 30 " | 14 |
| Between 1 day and 30 days, | 11 | " 30 " " 40 " | 15 |
| " 1 month and 6 months, | 11 | " 40 " " 50 " | 10 |
| " 6 months and 12 " | 13 | " 50 " " 60 " | 5 |
| " 1 year " 3 years, | 10 | " 60 " " 70 " | 4 |
| " 3 " " 5 " | 6 | " 70 " " 80 " | 6 |
| " 5 " " 10 " | 3 | " 80 " " 90 " | 0 |
| Total number under 10 years, | 64 | Total number over 10 years, | 56 |

Ages not given, 3. Total, 123

### NATIVITIES.

| | | | |
|---|---|---|---|
| American, | 48 | French, | 1 |
| German, | 37 | Norwegian, | 1 |
| Irish, | 12. | Canadian, | 1 |
| English, | 5 | African, | 2 |
| | | Country not given, | 16 |

Total, 123

